# Dynamics of Inflammatory Markers in Predicting Mortality in COVID-19

**DOI:** 10.7759/cureus.19080

**Published:** 2021-10-27

**Authors:** Aditi Parimoo, Ashutosh Biswas, Upendra Baitha, Gaurav Gupta, Shivam Pandey, Piyush Ranjan, Vandana Gupta, Debarchan Barman Roy, Bindoo Prakash, Naveet Wig

**Affiliations:** 1 Department of Medicine, All India Institute of Medical Sciences, New Delhi, IND; 2 Department of Biostatistics, All India Institute of Medical Sciences, New Delhi, IND; 3 Division of Periodontics, Center for Dental Education and Research, All India Institute of Medical Sciences, New Delhi, IND

**Keywords:** covid-19 india, outcome predictors in covid-19, mortality predictors, cytokine storm, inflammatory biomarkers

## Abstract

Introduction

A cytokine storm is an important cause of morbidity and mortality in patients with coronavirus disease 2019 (COVID-19). The objective of the study was to determine the prognostic significance of pro-inflammatory cytokines with the overall final outcome of patients with COVID-19.

Methods

We conducted a retrospective study of 142 patients admitted with COVID-19 in the Department of Medicine at All India Institute of Medical Sciences, New Delhi, from May 2021 to June 2021. We obtained their demographic, clinical, and biochemical characteristics at baseline and 48-72 hours prior to the terminal event (survival/death). The data were analyzed to determine the prognostic significance of these markers on the final outcome.

Results

Higher levels of inflammatory markers were associated with a worse final outcome (ferritin p-value <0.001, c-reactive protein (CRP) p-value <0.001, interleukin 6 (IL-6) p-value 0.007, procalcitonin p-value 0.005, and lactic acid p-value 0.004). Optimal probability cut-offs for these markers for predicting mortality were: ferritin: 963 ng/mL (sensitivity - 67.35%, specificity - 67.50%), CRP: 66.3 mg/L (sensitivity - 78.43%, specificity - 74.12%), IL-6: 46.2 pg/mL (sensitivity - 59.26%, specificity - 59.57%), procalcitonin: 0.3ng/mL (sensitivity - 65.38 %, specificity - 66.67%), lactic acid: 1.5 mg/dL (sensitivity - 59.26%, specificity - 58.57%). Multivariate logistic regression analysis was done, which showed that pre-terminal event CRP was associated with a statistically significant higher risk of mortality (Unadjusted OR 18.89, Adjusted OR 1.008, p=0.002, 95% CI 6.815 - 47.541).

Conclusion

Inflammatory markers have a prognostic significance in patients with COVID-19, with higher levels being associated with worse outcomes.

## Introduction

Coronavirus disease 2019 (COVID-19) is a raging pandemic that has affected millions of people worldwide. While most of the cases have an asymptomatic or mild course, a significant number of patients develop severe disease, often associated with a hyperinflammatory state called a cytokine storm [1]. Cytokine release syndrome is characterized by extensive tissue damage and end-organ dysfunction due to ineffective control of inflammatory cytokines released due to immune dysregulation [2]. This leads to a cytokine storm and a sudden rise in pro-inflammatory cytokines. It has been postulated that the effective lowering of pro-inflammatory cytokines is essential to control the hyperinflammatory state in severe COVID-19 and may be associated with improved clinical outcomes [3].

Cytokine storms manifest clinically with rapid clinical deterioration characterized by coagulopathy, hypotension, and multiple end-organ dysfunction [4]. Several markers of clinical severity have been proposed for the early identification of patients developing a cytokine storm and for their subsequent monitoring and prognostication [5].

Anti-inflammatory therapies directed at reducing the levels of these cytokines may be associated with better clinical outcomes. Therefore, we conducted a retrospective study to determine the correlation of the trend of pro-inflammatory cytokines, including ferritin, C-reactive protein (CRP), interleukin 6 (IL-6), procalcitonin, and lactic acid with the overall outcome of patients with COVID-19.

## Materials and methods

Study design

This was a retrospective observational study of 142 patients admitted with COVID-19 in the Department of Medicine at All India Institute of Medical Sciences, New Delhi, from May 2021 to June 2021. The study included all patients who were 18 years or older, with severe acute respiratory syndrome coronavirus 2 (SARS-CoV-2) infection confirmed by real-time reverse-transcription-polymerase chain reaction (RT-PCR) assay of nasal, pharyngeal, or lower respiratory tract samples. The study protocol was approved by the Ethics Committee of the All India Institute of Medical Sciences, New Delhi, with the IEC number: IEC-295/17.04.2020, RP-37/2020. We extracted the medical records and charts of each patient and reviewed all the data. Since the study was a retrospective study and did not hamper patients’ privacy, informed consent was waived.

Clinical and biological data

In this study, medical history, demographic characteristics, comorbidities, illness severity on admission, and laboratory findings were collected.

Laboratory investigations, including blood counts and liver and kidney function tests, were obtained at baseline. Serum ferritin, CRP, IL-6, procalcitonin, and lactic acid were collected at baseline and 48-72 hours prior to the final outcome of patients, and their change from baseline values was also noted. The primary outcome was patient survival during hospitalization. The final outcome of these admitted patients was categorized as either survivor or non-survivor.

Statistical analysis

Statistical analysis was conducted to determine the prognostic significance of these markers on the final outcome (survival or non-survival) using StataCorp. 2019 (Stata Statistical Software: Release 16. College Station, TX: StataCorp LLC). All categorical variables were expressed as frequency and percentage. All continuous variables were expressed as mean and standard deviation or median and range. Parameters of the individuals among survivors and non-survivors were compared using the chi-square test or Fisher’s exact test for categorical variables, independent t-test for continuous variables following normal distribution, and Wilcoxon rank-sum test for parameters following non-normal distribution. Receiver operating characteristic (ROC) curves were used to obtain the optimal probability cut-off to predict the final outcome of these markers. A p-value of <0.05 was considered significant.

## Results

Demographic and clinical features

A total of 142 patients were included in the study and their demographic characteristics and clinical features are described in Table 1. The mean age of non-survivors was higher than the mean age of survivors (55.67 + 17.41 years vs 49.50 + 15.36 years) and the difference was statistically significant (p= 0.01).

**Table 1:** Baseline characteristics and laboratory investigations of survivors and non-survivors *Hb: Hemoglobin, TLC: Total Leucocyte Count, AST: Aspartate Transaminase, ALT: Alanine Transaminase, ALP: Alkaline Phosphatase ^##^ p-value calculated using the independent t-test ** p-value calculated using Fisher's exact test ^#^ p-value calculated using Wilcoxon's Rank-Sum Test

VARIABLES	NON-SURVIVORS (n = 55)	SURVIVORS (n = 87)	p-VALUE
Age (in years)^##^ (Mean + S.D)	55.67 + 17.41	49.50 + 15.36	0.013
Male (n) (%age)**	33 (60)	48 (55.17)	0.32
Diabetes Mellitus (n) (%age)**	23 (41.82)	29 (33.33)	0.307
Hypertension (n) (%age)**	31 (56.36)	27 (31.03)	0.003
Coronary Artery Disease (n) (%age)**	2 (3.64)	5 (5.75)	0.587
Chronic Kidney Disease (n) (%age)**	4 (7.27)	4 (4.60)	0.501
Hb (g%)* ( Mean + S.D.)^#^	11.28 + 2.6	11.69 + 2.7	0.37
TLC (per cumm)* [Med (Range)]^#^	11,525 (730 – 71,500)	10,210 (370 – 49,200)	0.18
Total Bilirubin (mg%) [Med (Range)]^#^	0.50 (0.21 – 23.7)	0.55 (0.16 – 9.4)	0.65
AST (U/L)* [Med (Range)]^#^	41 (12 – 284)	40.5 (10 – 1649)	0.69
ALT (U/L)* [Med (Range)]^#^	30 (11 – 674)	40 (5 – 1562)	0.13
ALP (U/L)* [Med (Range)]^#^	103.5 (50 – 448)	102.5 (42 – 744)	0.91
Serum Urea (mg%) [Med (Range)]^#^	36.5 (10 – 160)	38 (3.8 – 336)	0.21
Serum Creatinine (mg%) [Med (Range)]^#^	0.8 (0.06 – 5.4)	0.8 (0.06 – 17.3)	0.21

Among the non-survivors, hypertension was present in 31 (56.36%) patients while amongst the survivors, it was present in 27 (31.03%) patients, and this difference amongst the two groups was statistically significant (p = 0.003).

Laboratory investigations

The laboratory investigations of survivors and non-survivors are described in Table 1.

Inflammatory biomarkers

In the study, the values of inflammatory markers among non-survivors and survivors and compared their median values among the two groups are shown in Table 2. The receiver operating characteristic (ROC) curve for each of the markers and obtained optimal probability cut-offs for the markers for predicting the final outcome of patients as shown in Table 3.

**Table 2:** Inflammatory markers among survivors and non-survivors *CRP: C-Reactive Protein, IL-6: Interleukin-6 # p-value calculated using Wilcoxon's rank-sum test

Variable	NON-SURVIVORS (n = 55)	SURVIVORS (n = 87)	P-VALUE
Ferritin (baseline) (ng/mL) [Med (Range)]	1009 (69.2 – 7580)	659 (25.93 – 5237)	<0.001
Ferritin (pre-terminal event) (ng/mL) [Med (Range)]	1395 (35.5 – 4825)	600.5 (28.1 – 5237)	<0.001
Ferritin (change from baseline) (ng/mL) [Med (Range)]	44 (-6855 – 3879)	-0.4 (-2242 – 3277)	0.07
CRP* (baseline) (mg/L) [Med (Range)]	102.45 (2.3 – 342.1)	37.94 (0 – 404.1)	<0.001
CRP (pre-terminal event) (mg/L) [Med (Range)]	116.8 (10.5 – 426)	14.38 (0 – 374.2)	<0.001
CRP (change from baseline) (mg/L) [Med (Range)]	0 (-308.2 – 300.1)	0 (-333.9 – 192.48)	0.13
IL-6* (pg/mL) [Med (Range)]	53.84 (11.24 – 783.32)	37.94 (0 – 1634)	0.007
Procalcitonin (ng/mL) [Med (Range)]	0.73 (0.04 – 45.4)	0.15 (0.02 – 29.8)	0.005
Lactic acid (mmol/L) [Med (Range)]	1.70 (0.8 – 5.9)	1.30 (0.5 – 7.3)	0.004

**Table 3:** Optimal probability cut-off, sensitivity, and specificity of inflammatory markers in predicting mortality CRP: C-Reactive Protein; IL-6: Interleukin 6

VARIABLES	OPTIMAL PROBABILITY CUT-OFF	SENSITIVITY (%AGE)	SPECIFICITY (%AGE)	AREA UNDER CURVE
Ferritin (ng/mL)	963	67.35	67.50	0.732
CRP (mg/L)	66.7	78.43	74.12	0.825
IL-6 (pg/mL)	46.2	59.26	59.57	0.686
Procalcitonin (ng/mL)	0.3	65.38	66.67	0.712
Lactic Acid (mmol/L)	1.5	59.26	58.57	0.647

The median ferritin level at baseline among non-survivors was 1009 ng/mL (69.2, 7580) as compared to 659 ng/ml (25.93, 5237) among survivors and this difference was statistically significant (p-value <0.001). The median ferritin level prior to the terminal event (survival or death) among non-survivors was 1395 ng/mL (35.5, 4825) as compared to 600.5 ng/mL (28.1, 5237) among survivors and this difference was statistically significant (p-value <0.001). The median value of change in ferritin level from baseline to the pre-terminal event among non-survivors was 44 ng/mL (-6855, 3879), whereas, among survivors, it was -0.4 ng/mL (-2242,3277). However, this difference was not statistically significant (p = 0.07).

Hence, higher ferritin levels prior to the terminal event (survival or death) and at baseline, were associated with a greater probability of an adverse outcome (p <0.001 and p <0.001, respectively). Ferritin levels prior to the terminal event were more strongly correlated with death than ferritin levels at baseline. The change in ferritin values during the course of hospital admission, however, was not associated with the final outcome of patients.

**Figure 1:**
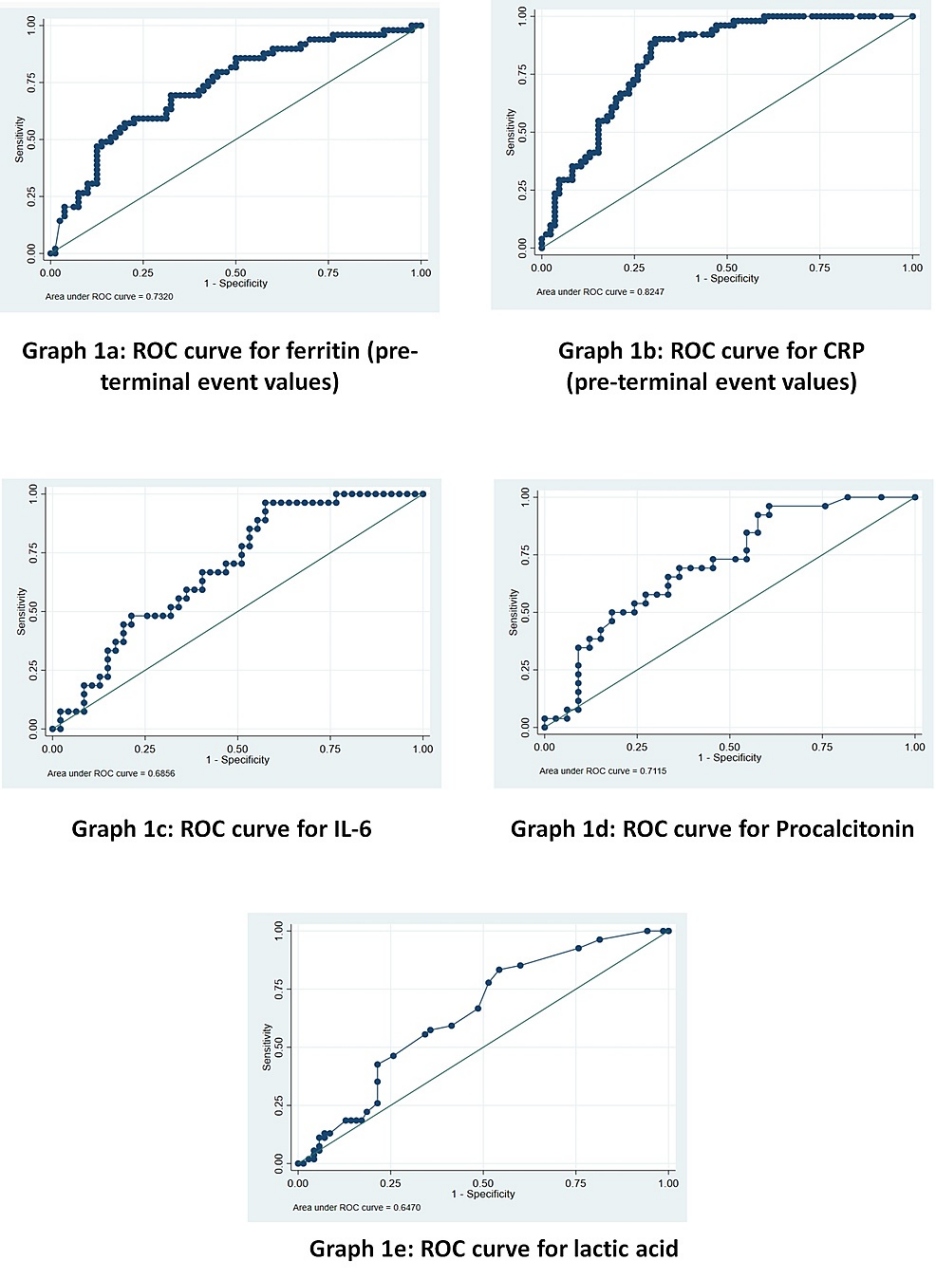
ROC curve assessing the predictive capability of various inflammatory biomarkers for death 1a: ROC curve for ferritin (pre-terminal event values), 1b: ROC curve for CRP (pre-terminal event values), 1c: ROC curve for IL-6, 1d: ROC curve for procalcitonin, 1e: ROC curve for lactic acid ROC: Receiver Operating Characteristic

The optimal probability cut-off for ferritin for predicting the final outcome prior to the terminal event was calculated using the ROC curve and came out to be 963 ng/mL, with a sensitivity of 67.35% and specificity of 67.50% in predicting the final outcome, that is, survival or death of patients (Figure 1a).

The median CRP level at baseline among non-survivors was 102.45 mg/L (2.3, 342.1) as compared to 37.94 mg/L (0, 404.1) among survivors and this difference was statistically significant (p <0.001). The median CRP level prior to the terminal event (survival or death) among non-survivors was 116.8 mg/L (10.5, 426) as compared to 14.38 mg/L (0, 374.2) and this difference was statistically significant (p <0.001). The median value of change in CRP level from baseline to the pre-terminal event among non-survivors was 0 (-308.2, 300.1) and 0 (-333.9, 192.48) and the p-value was statistically insignificant (p = 0.13). Hence, higher CRP levels prior to the terminal event and those obtained at baseline were associated with a greater probability of death (p <0.001 and p <0.001, respectively). The association was stronger for CRP levels prior to the terminal event than CRP levels at admission. The change in CRP values during the course of hospital admission was not associated with the final outcome of the patients.

Optimal probability cut-off for CRP prior to the terminal event for predicting the final outcome was calculated using the ROC curve and came out to be 66.7 mg/L with a sensitivity of 78.43% and specificity of 74.12% in predicting the final outcome, that is, survival or death of patients (Figure 1b).

The median IL-6 level among non-survivors was 53.84 pg/mL (11.24, 783.32) as compared to 37.94 pg/mL (0, 1634) among survivors. Hence, higher IL-6 levels were significantly associated with an adverse final outcome (p = 0.007).

The optimal probability cut-off for IL-6 was calculated using the ROC curve and came out to be 46.2 pg/mL with a sensitivity of 59.26% and a specificity of 59.57% in predicting the final outcome, that is, survival or death of patients (Figure 1c).

The median procalcitonin level among non-survivors was 0.73 ng/mL (0.04, 45.4) while the median procalcitonin level amongst survivors was 0.15 ng/mL (0.02, 29.8). Hence, higher procalcitonin levels were significantly associated with an adverse outcome (p = 0.005).

Optimal probability cut-off for procalcitonin for predicting the final outcome was calculated using the ROC curve and came out to be 0.3 ng/mL with a sensitivity of 65.38% and specificity of 66.67% in predicting the final outcome, that is, survival or death of patients (Figure 1d).

The median lactic acid level among non-survivors was 1.70 mmol/L (0.8, 5.9) as compared to 1.30 mmol/L (0.5,7.3). Hence, higher lactic acid values were significantly associated with a worse final outcome (p = 0.004).

The optimal probability cut-off for lactic acid for predicting the final outcome was calculated using the ROC curve and came out to be 1.5 mmol/L with a sensitivity of 59.26% and specificity of 58.57% in predicting the final outcome, that is, the survival or death of patients (Figure 1e).

Multivariate logistic regression analysis was done for the pre-terminal event CRP, pre-terminal event ferritin, lactic acid, age, and hypertension. A total of 113 observations were available for the multivariate analysis. However, due to the small sample size for patients included in the multivariate analysis, only pre-terminal event CRP was associated with a statistically significant higher risk of mortality (Unadjusted OR 18.89, Adjusted OR 1.008, p = 0.002, 95% CI 6.815 - 47.541). No other variables were associated with a statistically significant higher risk of mortality in COVID-19 disease as shown in Table 4.

**Table 4:** Logistic regression model for multivariate analysis of variables amongst survivors and non-survivors CRP: C-Reactive Protein

VARIABLES	UNADJUSTED ODD'S RATIO	ADJUSTED ODD'S RATIO	P-VALUE	95% CI
CRP	18.89	1.008	0.002	6.815 - 47.541
Ferritin	4.20	1.000	0.082	1.965 - 9.016
Lactic Acid	2.40	1.13	0.521	1.155 - 4.967
Age	2.65	0.999	0.977	1.310 - 5.363
Hypertension	2.87	2.119	0.153	1.425 - 5.781

## Discussion

We conducted a retrospective analysis of 142 COVID patients. We found that inflammatory markers, including ferritin, CRP, IL-6, procalcitonin, and lactic acid, were significantly raised in non-survivors as compared to survivors. Several studies have been conducted to ascertain the relationship between these markers and the overall outcome of patients with COVID-19 disease. Inflammatory markers have been reported to be predictors of mortality in patients with COVID-19 disease [6]. The cytokine storm due to the release of pro-inflammatory factors, akin to that reported in other infections, has been attributed to be a major cause of death in patients with severe COVID-19 disease [7]. A definite decline in the level of inflammatory markers was observed among survivors within 10 days post-admission among patients with moderate and severe coronavirus disease, whereas no significant reduction was seen in patients who died eventually in the study cohort [8].

Our study showed that high serum ferritin values prior to the terminal event (that is, survival or death) were more significantly associated with death as the final outcome than hyperferritinemia at baseline or the absolute change in serum ferritin values during the course of hospital admission with respect to the baseline values. High serum ferritin values at the time of admission have been independently associated with a severe disease course [9]. Hyperferritinemia has also been associated as an independent risk factor for acute respiratory distress syndrome (ARDS) in COVID-19 [10]. However, some reports have indicated that while hyperferritinemia is associated with a more severe course of the disease, it may not be associated with a worse prognosis [11]. The findings of our study are in contrast with these earlier reports since high serum ferritin values, particularly prior to the terminal event have been significantly associated with an adverse final outcome.

Similarly, it was observed in our study that higher CRP levels prior to the terminal event (survival or non-survival) were more significantly associated with an adverse final outcome than the CRP values obtained at baseline and the absolute change in CRP values from baseline during the course of hospital admission. Earlier reports have also indicated that CRP levels at the time of admission and prior to discharge or death are markers of poor prognosis in patients with COVID-19 [12]. Higher CRP values during the initial stages of the disease were associated with greater CT severity scores and extensive lung involvement [13]. Higher CRP values have been positively correlated with increased mortality in COVID-19 patients in similar study cohorts [14].

We observed that IL-6 was significantly higher amongst non-survivors as compared to survivors in our study group. These findings reiterate the fact that COVID-19 is a hyperinflammatory state associated with an increase in pro-inflammatory cytokines and raised inflammatory markers are associated with a severe disease course and an adverse final outcome as suggested by earlier studies [14-16].

In our study, we observed that higher procalcitonin levels were associated with a worse prognosis. These findings are in concordance with earlier studies reported in the literature [16]. Elevated procalcitonin levels were associated with increased mortality as assessed by subgroup analyses conducted in a meta-analysis of inflammatory markers for predicting prognosis in COVID-19 patients [17].

We also observed that elevated lactic acid values were associated with a worse overall outcome of patients. This has also been suggested by other studies reported in the literature [18]. Lactic acidosis may itself contribute to higher mortality in patients with severe COVID-19 [19].

All the variables with a significant p-value on univariate analysis were then analyzed using the Cox regression model. However, only pre-terminal event CRP was significantly associated with a worse overall outcome. This may be attributed to the very small sample size available for multivariate analysis.

The limitations of our study included the small sample size and the retrospective nature of our study. The effect of various therapies, including anti-inflammatory drugs, and other supportive therapies, such as antibiotics, vasopressors, etc., also could not be studied, as this was a retrospective study and, hence, precluded the randomization of study participants to different treatment protocols.

## Conclusions

Inflammatory markers, including ferritin, CRP, IL-6, procalcitonin, and lactic acid, have prognostic significance in patients with COVID-19 disease. Higher levels of these inflammatory markers, particularly later during the course of hospital admission, are associated with a higher likelihood of non-survival in these patients. An increase in these inflammatory markers is an early indicator of cytokine storm in these patients, which is a harbinger of a relatively poor prognosis and is associated with higher mortality in patients with COVID-19. This will help clinicians in the early identification of high-risk patients. Therefore, measuring the levels of these inflammatory markers early in the disease helps in prognosticating patient outcomes. This, in turn, helps in tailoring early, appropriate, efficient, and effective treatment of patients, which will lead to a significant reduction in mortality related to COVID-19.
